# Unveiling transcriptional mechanisms of B7-H3 in breast cancer stem cells through proteomic approaches

**DOI:** 10.1016/j.isci.2025.112218

**Published:** 2025-03-14

**Authors:** Yu Ri Seo, Han Byeol Kim, Hyeryeon Jung, Eunhee G. Kim, Sumin Huh, Eugene C. Yi, Kristine M. Kim

**Affiliations:** 1 Department of Molecular Medicine and Biopharmaceutical Sciences, Graduate School of Convergence Science and Technology and College of Medicine or College of Pharmacy, Seoul National University, Seoul, Republic of Korea; 2 Institute of Medical and Biological Engineering, Seoul National University Medical Research Center, Seoul, Republic of Korea; 3 Department of Bio-Health Convergence, Kangwon National University, Chuncheon, Republic of Korea; 4 Department of Systems Immunology, Division of Biomedical Convergence, College of Biomedical Science, Kangwon National University, Chuncheon, Republic of Korea

**Keywords:** molecular biology, cell biology, cancer, omics

## Abstract

B7-H3, an immune checkpoint molecule, is prominently overexpressed in various solid tumors, correlating with poor clinical outcomes. Despite its critical role in promoting tumorigenesis, metastasis, and immune evasion, the regulatory mechanisms governing B7-H3 expression, particularly in cancer stem cells (CSCs), remain elusive. In this comprehensive study, we focused on breast CSCs to uncover the transcriptional regulators driving B7-H3 overexpression. Utilizing DNA affinity purification-mass spectrometry (DAP-MS) to analyze B7-H3 promoter regions, we identified a novel set of transcription factors, including DDB1, XRCC5, PARP1, RPA1, and RPA3, as key modulators of B7-H3 expression. Functional assays revealed that targeting DDB1 with nitazoxanide significantly downregulated B7-H3 expression, subsequently impairing tumor sphere formation and cell migration in breast CSCs. These findings not only elucidate the complex transcriptional network controlling B7-H3 expression but also open new avenues for developing targeted immunotherapies aimed at disrupting CSC-driven cancer progression.

## Highlights

• Key transcription factors of B7-H3 in breast CSCs are identified using DAP-MS
• DDB1, XRCC5, PARP1, RPA1, and RPA3 regulate B7-H3 expression in breast CSCs
• Nitazoxanide inhibits DDB1, reducing B7-H3 expression and CSC properties
• Insights into B7-H3 regulation suggest new CSC-targeted therapeutic strategies

## Graphical abstract

**Figure:**
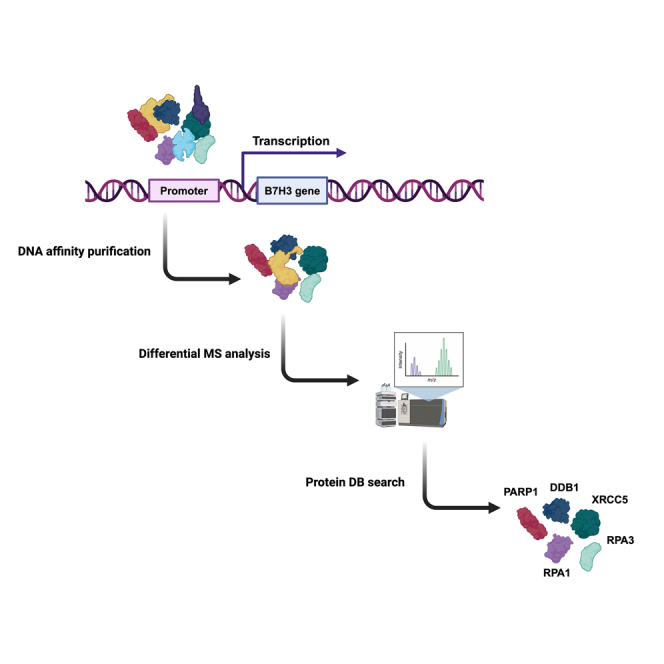

## Introduction

B7-H3, a type I transmembrane glycoprotein and member of the B7 superfamily, plays a pivotal role in immune regulation, influencing T-lymphocyte activity through either co-stimulatory or co-inhibitory effects on the T cell immune response.^1^ Studies suggest that B7-H3 can enhance T cell responses and potentially boost anti-tumor immunity.^2^^,^^3^ Conversely, other research indicates that B7-H3 primarily functions as a co-inhibitory molecule, suppressing T cell activity and facilitating immune evasion by tumors.^4^^,^^5^ This inhibitory role is hypothesized to promote tumor progression by enabling cancer cells to evade immune surveillance.

B7-H3 is widely overexpressed in various human solid cancers, including breast cancer, and its high expression often correlates with poor clinical outcomes.^5^^,^^6^ This overexpression suggests that B7-H3 might contribute to tumorigenesis and metastasis by modulating the tumor microenvironment and immune response.^2^^,^^7^^,^^8^ Additionally, recent studies have highlighted B7-H3’s role beyond immune modulation, particularly in cancer stem cells (CSCs), where it is implicated in maintaining stemness properties such as self-renewal, tumorigenicity, and metastatic potential.^5^^,^^9^^,^^10^ However, the exact mechanisms through which B7-H3 influences these processes remain poorly understood.

A study suggests that B7-H3 expression may be regulated by a post-transcriptional mechanism, as evidenced by the discrepancies observed between its mRNA and protein expression patterns.^2^^,^^11^ Although B7-H3 mRNA is widely expressed in most human tissues, the B7-H3 protein is not ubiquitously present, suggesting the potential for stringent post-transcriptional control.^12^^,^^13^ MicroRNAs (miRNAs) appear to play a significant role in regulating B7-H3 in various solid tumors. For example, miR-124 targets the 3ʹ-UTR of B7-H3, functioning as a tumor s suppressor.^14^ Similarly, miR-29 inhibits B7-H3 to suppress medulloblastoma angiogenesis,^11^ and miR-128 negatively regulates B7-H3 expression in colorectal cancer.^15^

In addition to miRNAs, several transcription factors regulate B7-H3 expression. SP1 binds to the promoter region of B7-H3, enhancing its transcription in certain cancer cells.^16^ Furthermore, the interplay between hypoxia-inducible factor 1 alpha (HIF-1α) and B7-H3 has been observed, where HIF-1α upregulates B7-H3 expression under hypoxic conditions, contributing to the adaptation of cancer cells to low oxygen environments.^11^^,^^17^ Another important regulatory factor is STAT3, which has been shown to modulate B7-H3 expression through the JAK/STAT signaling pathway, particularly in cancers with high STAT3 activity.^18^^,^^19^ Additionally, nuclear factor κB (NF-κB), a key regulator of inflammatory responses, can also influence B7-H3 expression, linking inflammation and immune evasion mechanisms in tumors.^20^^,^^21^

Given B7-H3’s significant overexpression in cancers, particularly in breast cancer, there has been growing interest in developing therapies that specifically target this molecule. However, the exact immune modulatory mechanisms of B7-H3 remain elusive, mainly due to the unidentified ligand receptor, making it unclear whether B7-H3 acts as a co-inhibitor or co-activator in immune responses. As a result, current therapeutic approaches, such as antibody-drug conjugates (ADCs), have been developed not based on B7-H3’s intrinsic immune regulatory functions but rather solely on its overexpression in tumor cells. These strategies aim to exploit B7-H3’s high expression levels to deliver cytotoxic agents directly to cancer cells while sparing normal tissues.^22^^,^^23^^,^^24^^,^^25^^,^^26^

Previously, we demonstrated that B7-H3 protein is significantly overexpressed in breast CSC-like cells compared to non-CSC (NCSC)-like cells derived from MDA-MB453, as revealed through cell-surface proteome analysis.^27^ However, the precise mechanisms driving B7-H3 upregulation in CSCs remain largely unexplored. Despite these findings, further comprehensive studies are necessary to fully elucidate the underlying regulatory pathways governing B7-H3 expression.

In this study, we sought to uncover the transcription factors (TFs) responsible for the overexpression of B7-H3 in breast CSC-like cells. Through DNA affinity purification (DAP) combined with mass-spectrometry-based proteomic analysis,^28^ we identified several key proteins binding to the B7-H3 promoter. Subsequent validation through gene knockdown experiments and DNA binding assays revealed that XRCC5, PARP1, DDB1, RPA1, and RPA3 play critical roles in regulating B7-H3 expression. Knockdown of these TFs significantly diminished hallmark CSC characteristics, including tumor sphere formation and cell migration. Among these, DDB1 emerged as the most significant transcriptional regulator of B7-H3. By treating CSCs with nitazoxanide, a known inhibitor of DDB1, we observed a marked reduction in B7-H3 expression, alongside diminished tumor sphere formation and migration capacity. These findings illuminate the regulatory mechanisms of B7-H3 overexpression in CSCs and suggest that targeting transcription factors involved in B7-H3 regulation may represent a promising approach for developing novel cancer therapies.

## Results

### B7-H3 is highly expressed in CSC-like cell populations

**Figure 1:**
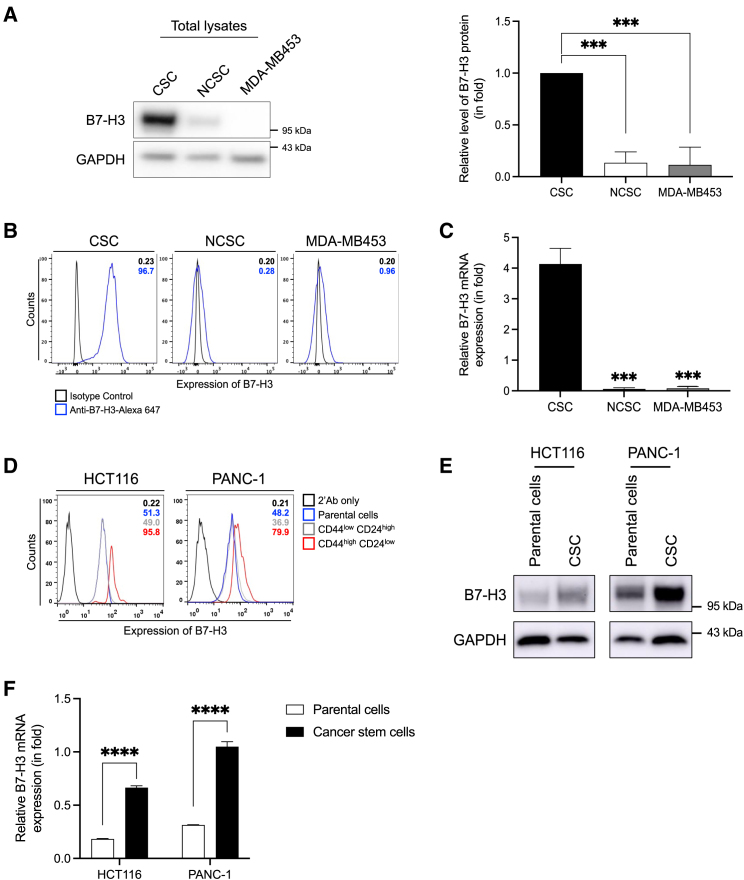
B7-H3 expression in CSCs populations (A) Western blot analysis of B7-H3 in breast cancer CSCs, NCSCs, and parental MDA-MB453 cells. GAPDH was used as a loading control. (B) Flow cytometry analysis of B7-H3 expression in breast cancer CSCs, NCSCs, and MDA-MB453 (parental cells). Histograms show B7-H3 staining (blue line) versus isotype control IgG1κ (black line). (C) Quantitative RT-PCR analysis of B7-H3 in breast cancer CSCs, NCSCs, and parental MDA-MB453 cells. β-actin mRNA was used for normalization. (D) B7-H3 expression levels in CSCs population (CD44^high^CD24^low^), NCSCs population (CD44^low^CD24^high^), and their respective parental cells in HCT116 and PANC-1 cell lines. Histograms show B7-H3 staining (blue line) versus secondary Ab only (black line). B7-H3 expression was assessed by (E) western blot and (F) quantitative RT-PCR in CSC-eniched populations (CSC-HCT116 and CSC-PANC-1) and their parental cell lines (HCT116, PANC-1). Representative results from three independent experiments are shown. All values are expressed as mean ± SD. ∗*p* < 0.05, ∗∗*p* < 0.01, ∗∗∗*p* < 0.001, and ∗∗∗∗*p* < 0.0001.

The association between CSC and B7-H3 expression has been previously described.^27^ However, the precise mechanisms underlying the upregulation of B7-H3 expression in CSCs remain unclear. To investigate this further, we verified the expression level of B7-H3 in CSCs. Flow cytometry and western blot analyses showed significantly higher expression of B7-H3 in CSCs compared to both NCSCs and their parental MDA-MB453 cell lines (Figures 1A and 1B). Quantitative RT-PCR analysis also revealed that B7-H3 mRNA levels were increased in CSCs compared to NCSCs and their parental cells (Figure 1C).

In order to further evaluate the specificity and persistence of B7-H3 expression in CSCs populations across different cancer types, we analyzed B7-H3 expression in CD44^high^CD24^low^ CSCs populations from PANC-1 and HCT116 cell lines using flow cytometry. All CSCs populations (CD44^high^CD24^low^) exhibited high B7-H3 expression compared with NCSCs populations (CD44^low^CD24^high^) or their parental cells (Figure 1D). Furthermore, we isolated CSCs populations from HCT116 and PANC-1 cell lines using a sphere culture system and subsequently analyzed B7-H3 expression levels through western blot and RT-qPCR. Both protein or mRNA levels of B7-H3 were notably upregulated in these CSCs populations compared to their parental cells (Figures 1E, 1F, and S1), suggesting a functional association between B7-H3 and CSC-like properties.

### Identifying B7-H3 promoter DNA binding proteins using DNA affinity purification-mass spectrometry

Given that CSCs, including the CD44^high^CD24^low^-enriched populations of various types of cancer cell, exhibit a substantial increase in B7-H3 expression, we aimed to identify the TFs responsible for regulating B7-H3 expression. First, we performed a DNA sequence homology analysis for the region upstream of the transcription start site (TSS) of the human B7-H3 gene, which is located 418 bp upstream (5′) from exon1, using Clustal Omega of EMBL-EBI (https://ebi.ac.uk/Tools/msa/clustalo). Our analysis revealed that the sequence from the TSS to approximately 1,000 bp upstream is well conserved among humans, rats, and mice (Figure S2). This suggests that the conserved sequence is a crucial regulatory region for B7-H3 transcription.

**Figure 2:**
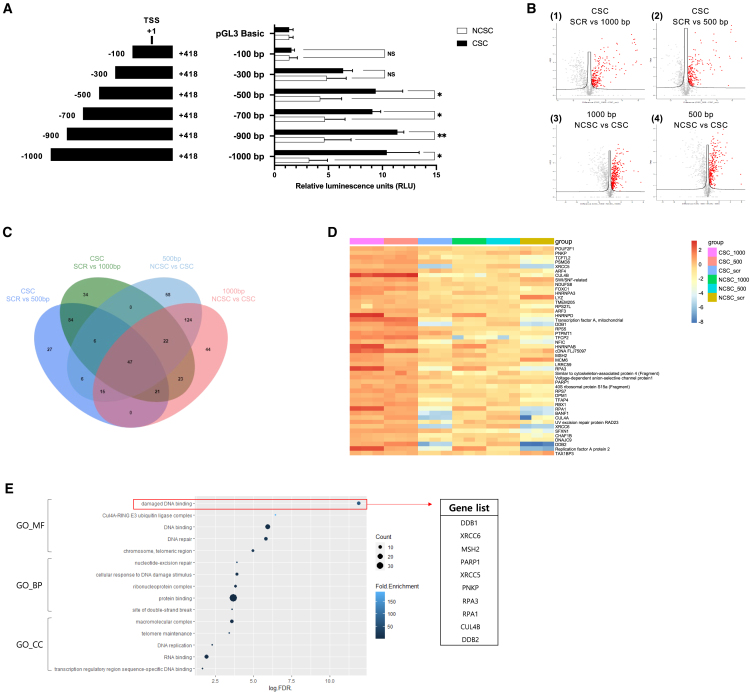
Proteomic analysis of B7-H3 promoter-binding proteins in CSCs and NCSCs (A) Dual luciferase reporter assays using truncated B7-H3 promoter constructs in CSCs and NCSCs. Promoter fragments of human B7-H3 were cloned into the pGL3 Basic vector, and relative luciferase activity was measured following transfection into CSCs and NCSCs. The pGL3 Basic vector without an insert served as a negative control. Luciferase activity was normalized as the ratio of Firefly luciferase to Renilla luciferase. Representative results from three independent experiments are shown. Data are presented as mean ± SD. Statistical significance was determined as follows: ∗p < 0.05 and ∗∗p < 0.01. (B) Volcano plots of protein abundance between CSCs and NCSCs using 500 and 1,000 bp B7-H3 promoter DNA fragments against a 500 bp scrambled DNA control. Each dot represents a protein, black cutoff lines indicate q value <0.05. Red dots represent upregulated proteins, while gray dots represent downregulated proteins or non-significant proteins. Comparisions include: (1) 1,000 bp promoter vs. scrambled DNA in CSCs, (2) 500 bp promoter vs. scrambled DNA in CSCs, (3) CSCs vs. NCSCs using 1,000 bp promoter DNA, and (4) CSCs vs. NCSCs using 500 bp promoter DNA. (C) Venn diagram showing the overlap of significantly upregulated proteins across the four comparision groups. (D) The heatmap displays the *Z* scores of the 47 proteins across the four comparision groups. (E) Gene Ontology (GO) analysis of the 47 upregulated proteins. Only the top five significant enriched terms from each GO catagory are shown. Point size indicates the number of proteins associated with each term, and point color represents the fold enrichment score. The x axis displays the statistical significance of enrichment as Log10(FDR).

To identify the specific promoter region for CSC-dependent transcriptional activity of B7-H3, we cloned six different B7-H3 promoter fragments, ranging in size from 100 to 1,000 bp, upstream of the luciferase reporter gene in vector pGL3 Basic. As described in Figure 2A, conducting luciferase assays with these promoter constructs in CSCs and NCSCs revealed that the regulatory regions shortened to 500 bp upstream in CSCs showed higher luciferase activities compared with NCSCs, suggesting that the CSC-specific regulatory region responsible for B7-H3 transcription lies within from −300 bp to −500 bp upstream of the TSS.

To elucidate the potential TFs interacting with the identified promoter region, we conducted DNA affinity purification-mass spectrometry (DAP-MS) analysis using a biotinylated DNA probe. Biotinylated 500 bp and 1,000 bp DNA probes were prepared including a 500 bp scrambled DNA (SCR) fragment as a negative control, with 5′ ends modified by biotin. We isolated DNA co-purifying proteins by incubating nuclear extracts with DNA probes. We compared the datasets for CSC and NCSCs using 1,000 bp and 500 bp promoter DNA through DAP-MS. This was followed by volcano plot analysis (q value >0.05) based on label-free quantitative analysis to identify proteins bound to DNA. As shown in Figure 2B, the relative abundance of proteins between the two samples [log2(fold change), x axis] is plotted against statistical significance (Log *p* value, y axis), with each point representing a protein identified by two or more unique peptides. Using criteria of q value >0.05, we identified 237 and 206 DNA binding proteins in CSCs that are specific to the 500 bp and 1,000 bp DNA probes compared to negative control, respectively. We then further compared proteins bound to the 500 bp and 1,000 bp DNA probes in CSCs and NCSCs. With the same quantitative criteria, we identified 254 proteins specifically bind to the 1,000 bp promoter DNA and 278 proteins to the 500 bp promoter DNA in CSC lysate, compared to NCSC lysate. Finally, Venn diagrams analysis confirmed that 47 proteins bound more strongly to the B7-H3 promoter DNA in CSC lysate compared to NCSC lysate (Figure 2C). Heatmap analysis based on these 47 proteins confirmed that they bind significantly to the B7-H3 promoter DNA in CSC lysate compared to NCSC lysate (Figure 2D). These 47 proteins were then subjected to Gene Ontology (GO) analysis to determine functional enrichment (Figure 2E). Based on the results of GO analysis, we identified the GO term with the highest confidence according to FDR criteria. From this GO term, 10 proteins were selected as potential TF candidates for B7-H3. To further refine the list, we utilized the volcano plot analysis result (Figure 2B(3)) based on the DAP-MS results and selected seven proteins showing a fold change >1.5 (Figure S3). Among these seven proteins, we excluded Cul4B upon confirming that it does not directly bind to DNA. Transcription factors generally possess DNA binding domains that allow them to attach to specific DNA sequences and regulate transcription. Cul4B, however, lacks these crucial DNA binding domains and instead operates through interactions with other proteins. Rather than being a direct transcription factor, Cul4B serves as a co-factor and regulatory protein, influencing transcription indirectly by regulating the stability of specific transcription factors or regulatory proteins.^29^^,^^30^^,^^31^^,^^32^ Given its lack of direct DNA binding capability, Cul4B was excluded from our final list. Consequently, we finalized the selection of the following six potential TF candidates: DDB1 (UniProt ID: Q16531), DDB2 (UniProt ID: Q92466), XRCC5 (UniProt ID: P13010), PARP1 (UniProt ID: P09874), RPA1 (UniProt ID: Q95602), and RPA3 (UniProt ID: P35244).

### Characterizing the functions of transcription factor candidates for B7-H3

**Figure 3:**
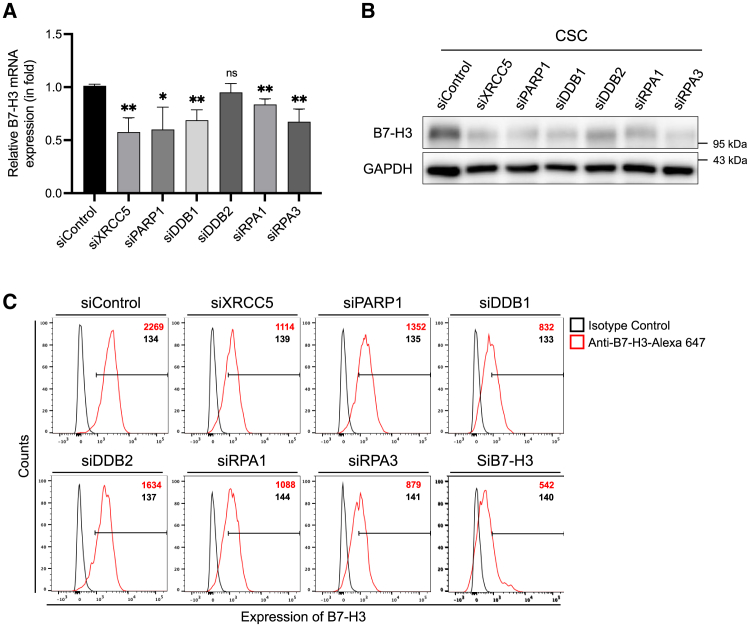
Regulation of B7-H3 expression by TFs in CSCs (A) mRNA expression levels of B7-H3 in CSCs following transfection with siRNA targeting candidate proteins (siXRCC5, siPARP1, siDDB1, siDDB2, siRPA1, and siRPA3), with siControl as a negative control and siB7-H3 as a positive control. Representative results from three independent experiments are shown. All values are expressed as mean ± SD. ∗p < 0.05 and ∗∗p < 0.01. (B) Protein expression levels of B7-H3 following siRNA-mediated knockdown of candidate TFs were assessed by Western blot, with GAPDH used as a loading control. (C) Flow cytometry analysis of B7-H3 expression following siRNA-mediated knockdown of candidate proteins. Histograms show B7-H3 staining (red line) compared to isotype control IgG1κ (black line).

In order to validate the identified six potential TF candidates, we generated CSC lines with targeted knockdowns for each TF candidate and subsequently measured B7-H3 expression levels under each knockdown condition by western blot, quantitative RT-PCR, and flow cytometry analysis (Figures 3A–3C). Based on the results of these knockdown experiments, we were able to exclude DDB2, which did not affect B7-H3 expression, and select the remaining five proteins—XRCC5, PARP1, DDB1, RPA1, and RPA3—as the final newly identified TFs for B7-H3. To further validate these findings, we extended our analysis to include HCT116 and HeLa cell lines with the same TF knockdowns. Consistent with our observations in CSCs, knockdown of these TFs in both HCT116 and HeLa cell lines also led to reduced levels of B7-H3 protein and mRNA, reinforcing their regulatory roles in the expression of B7-H3 (Figure S4).

**Figure 4:**
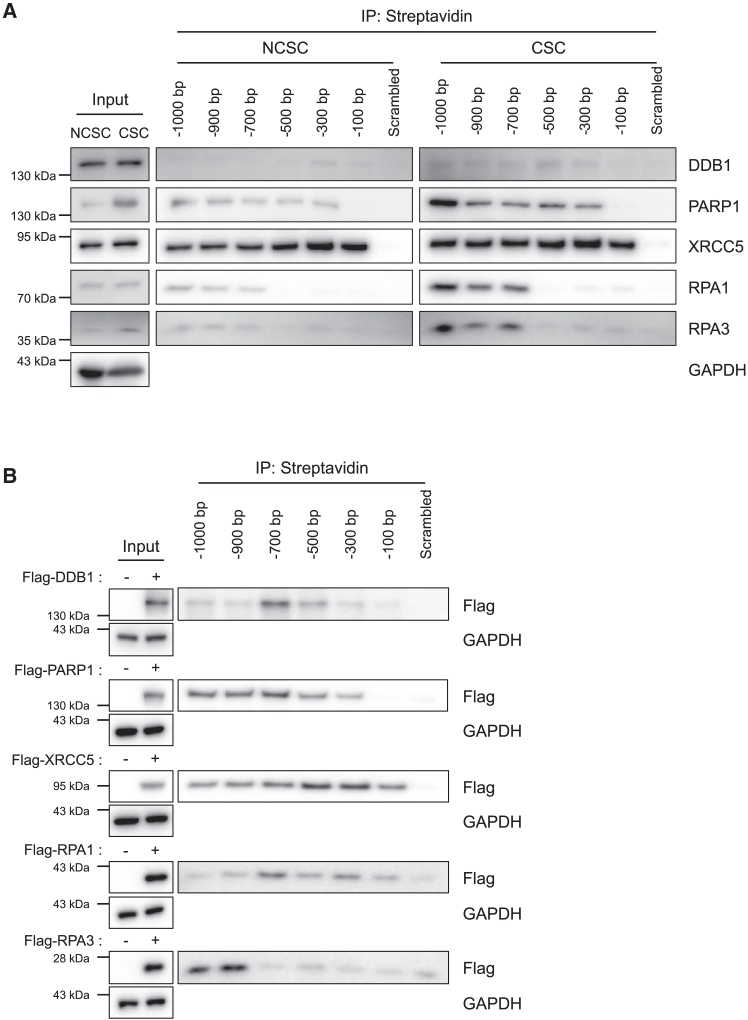
Verification of TFs binding to the B7-H3 promoter using a DNA pull-down assay Biotinylated B7-H3 promoter DNA fragments [-1,000 bp, -900 bp, -700 bp, -500 bp, -300 bp, and -100 bp relative to the TSS (+1)] were pulled down with streptavidin beads. A 500 bp scrambled DNA fragment was used as a negative control. (A) Western blot analysis confirming the binding of TFs to the B7-H3 promoter DNA in CSCs and NCSCs. Bands correpond to the endogenous levels of the five candidate TFs at thier predicted molecular weights. Total cell lysates (input) from each group were used as positive controls, and GAPDH was included as a loading control. (B) Straptavidin pull-down assay reveals the binding of TFs to the B7-H3 promoter DNA. Biotinylated promoter DNA fragments were incubated with lysates from HEK293 cells overexpressing FLAG-tagged TFs, and bound proteins were detected using an anti-FLAG antibody. Total cell lysates (input) were used as a positive control to confrim TF overexpression. GAPDH was included as a loading control.

To quantify the binding of the identified TFs to the B7-H3 promoter, we conducted an experiment using biotinylated DNA segments corresponding to different regions upstream of the transcription start site (TSS) at positions 1,000; 900; 700; 500; 300; and 100 bp. These segments were incubated with cell lysates from both CSC-like cells and NCSCs, with a DNA serving as a negative control. The binding of the TFs to each segment of the B7-H3 promoter was then assessed using western blot analysis. Our results indicated a significantly higher binding affinity of the TFs to the B7-H3 promoter in CSCs compared to NCSCs, despite similar overall TF expression levels in both cell types (Figure 4A). To further validate the binding interaction between the identified TFs and the B7-H3 promoter regions, we conducted additional experiments in HEK293 cells overexpressing FLAG-tagged versions of the TFs. These experiments confirmed the specific binding of the TFs to the B7-H3 promoter regions (Figure 4B).

Taken together, these findings corroborate the initial DAP-MS results and confirm the regulatory involvement of the selected five TFs in controlling B7-H3 expression. Our data distinctly illustrate that these TFs specifically bind to the B7-H3 promoter DNA, thereby substantiating their critical roles in the transcriptional regulation of B7H3 gene.

### Characterization transcription factors associated with cancer stemness

**Figure 5:**
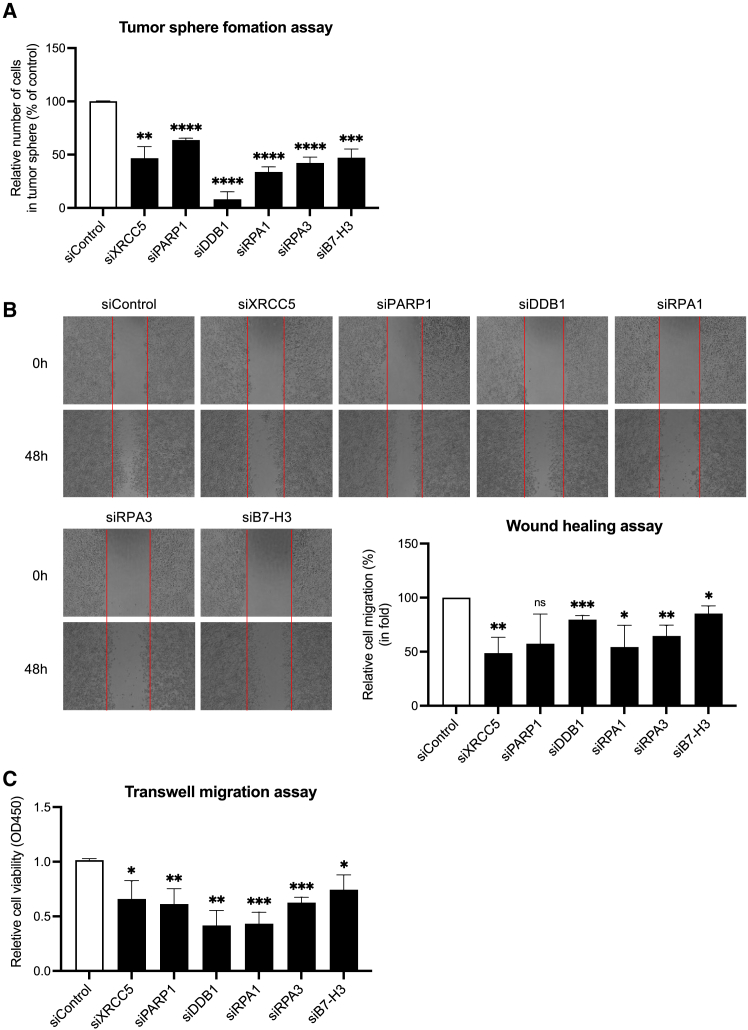
Characterization of TFs regulating CSC properties (A) Tumor sphere formation in CSCs following siRNA-mediated knockdown of DDB1, PARP1, XRCC5, RPA1, RPA3, and B7-H3, compared to cells treated with non-silencing control siRNA (siControl). (B) Wound-healing assay and (C) transwell migration assay performed in CSCs following siRNA-mediated knockdown of DDB1, PARP1, XRCC5, RPA1, RPA3, and B7-H3. Representative results from three independent experiments are shown. Data are presented as means ± SD. Statistical significance was determined as follows: ∗*p* < 0.05, ∗∗*p* < 0.01, ∗∗∗*p* < 0.001, and ∗∗∗∗*p* < 0.0001.

In addition to its co-inhibitory role of B7-H3 on T cells, recent evidence has shown that B7-H3 also plays a key role in promoting and maintaining cancer stemness, including drug resistance, self-renewal ability, and migration.^33^ To investigate the functional associations between cancer cell stemness and TFs that regulate B7-H3 expression, we utilized tumor-sphere formation and cell migration assays to access the role of B7-H3 in promoting stemness characteristics in CSC. As expected, CSCs demonstrated enhanced tumor sphere formation capabilities compared to NCSCs (Figure S5A). Additionally, knockdown of B7-H3 in CSCs led to a significant reduction in stemness traits, confirming B7-H3’s role as a regulator of CSC properties (Figure S5B). Further investigations revealed that knockdown of the TFs candidates—DDB1, PARP1, XRCC5, RPA1, and RPA3—resulted in a significant reduction in tumor sphere formation. This observation suggests that these TFs play a crucial role in maintaining CSC traits through the transcriptional regulation of B7-H3 (Figure 5A). Additionally, we assessed the influence of these TFs on CSC migration through wound healing and transwell migration assays. The results indicated that knockdown of these TFs significantly impaired cell migration in CSCs, further substantiating their role in regulating CSC properties (Figures 5B and 5C).

Upon comprehensive evaluation of these results, it was determined that among the TFs, DDB1 exhibited the most significant effect on the stemness characteristics of CSCs. This finding underscores the central role of DDB1 in the transcriptional regulation of B7-H3 and by extension its pivotal function of maintaining and promoting the defining traits of CSCs.

### DDB1 inhibitor reduces B7-H3 expression to suppress stemness properties of cancer stem cells

Building on our observations that DDB1 plays a critical role in regulating B7-H3 expression in CSCs, we hypothesized that targeting DDB1 with an inhibitor, nitazoxanide (NTZ), might effectively suppress B7-H3 expression and thereby influence the stemness properties of CSCs, including self-renewal, drug resistance, and migration.

**Figure 6:**
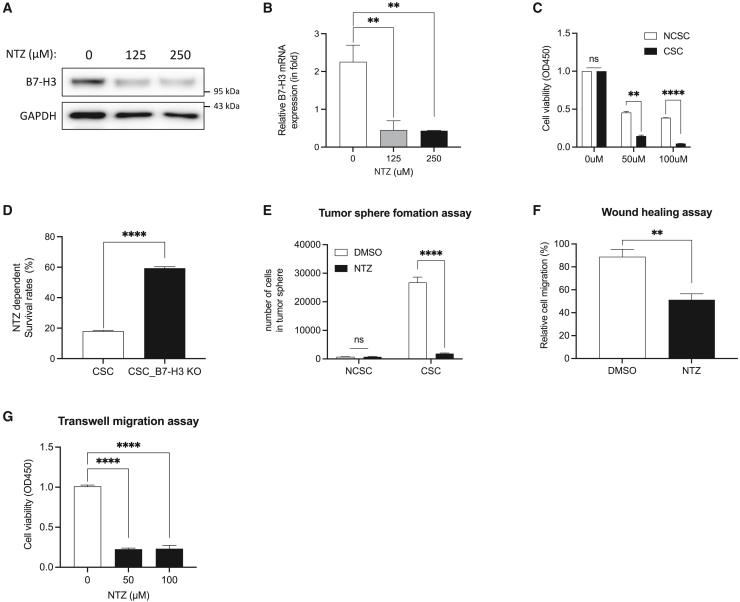
Effect of NTZ on B7-H3 expression and stemness properties in CSCs (A) B7-H3 protein and (B) mRNA expression levels in CSCs followingNTZ treatment. Western blot analysis was performed using GAPDH as a loading control, and quantitative RT-PCR was conducted with β-actin mRNA as the normalization control. (C) Cell viability assay of CSCs and NCSCs following NTZ treatment. (D) Comparison of NTZ-induced cytotoxicity between CSCs and B7-H3 knockout CSCs. (E) Tumor-sphere formation in CSCs and NCSCs following NTZ treatment. Cell migration assessment using (F) wound healing assay and (G) transwell migration assay to access cell migration in CSCs treated with NTZ. Representative results from three independent experiments are shown. Data are presented as mean ± SD. Statistical significance was determined as follows: ∗*p* < 0.05, ∗∗*p* < 0.01, ∗∗∗*p* < 0.001, and ∗∗∗∗*p* < 0.0001.

To assess the effect of NTZ on the expression of B7-H3, CSCs were treated with NTZ at concentrations of 125 and 250 μM. We observed a marked decrease in both protein and mRNA levels of B7-H3 by the treatment of NTZ (Figures 6A and 6B). Further investigations were conducted to determine the effect of NTZ on CSCs viability. CSCs treated with 50 μM NTZ demonstrated a significant reduction in cell viability compared to NCSCs (Figure 6C). Notably, B7-H3 knockout CSCs exhibited decreased sensitivity to NTZ treatment compared to the negative control, suggesting that reduced B7-H3 expression diminishes the sensitivity of CSCs to NTZ (Figure 6D). Given the identified role of B7-H3 in tumor formation and migration, we also assessed whether NTZ could impact these cellular functions in CSCs. Our results indicated that NTZ treatment significantly reduced both tumor sphere formation and cell migration (Figures 6E–6G), supporting the notion that NTZ affects CSCs characteristics by regulating B7-H3 expression through the inhibition of the DDB1 TF.

## Discussion

B7-H3, a well-known immune checkpoint molecule, is overexpressed in a variety of solid tumors and is often associated with poor clinical outcomes.^5^^,^^6^ This overexpression poses significant challenges in cancer treatment due to its role in promoting tumorigenesis, metastasis, and immune evasion.^2^^,^^7^^,^^8^ A major challenge is the lack of comprehensive understanding of the mechanisms that regulate B7-H3 expression, particularly in CSCs. Additionally, B7-H3’s ligand has not been identified, leaving its functional roles in immune modulation, whether as a co-inhibitor or co-activator, unclear.^2^^,^^16^^,^^34^ These complexities and heterogeneities have made it difficult to develop effective therapies targeting B7-H3 in cancers.

This lack of understanding about B7-H3’s precise functions has hindered the development of therapies that leverage its intrinsic immune regulatory roles. Consequently, current therapeutic strategies have focused on targeting the overexpression of B7-H3 in cancer cells rather than its immune modulatory functions. For example, antibody-drug conjugates have been developed to target B7-H3-expressing tumor cells,^22^^,^^23^^,^^24^^,^^25^ aiming to deliver cytotoxic agents specifically to cancer cells while sparing normal tissues. Despite these advancements, the lack of clarity regarding B7-H3’s precise role in immune modulation remains a significant hurdle, necessitating further research to optimize these therapies and fully exploit B7-H3 as a therapeutic target.

Elucidating the mechanisms underlying B7-H3 overexpression is crucial for developing targeted therapies.^2^^,^^4^^,^^35^ Identifying the TFs involved in regulating B7-H3 expression can provide insights into the pathways and processes that sustain CSC properties. By focusing on these TFs, it is possible to uncover potential therapeutic targets that could disrupt the regulatory networks maintaining CSCs, thus offering new avenues for cancer treatment.

In this study, we aimed to elucidate the regulatory mechanisms underlying the overexpression of B7-H3 in CSCs. We employed DAP-MS, a technique known for its effectiveness in identifying TFs when combined with quantitative proteomics methods. Despite its effectiveness, the DAP-MS method presents analytical challenges, primarily the difficulty in distinguishing between specific and non-specific proteins due to co-purified non-specific proteins during the immunoprecipitation (IP) process.^36^^,^^37^

To address these challenges and ensure the identification of specific components involved in B7-H3’s expression regulation, we implemented several strategies. Firstly, we conducted a systematic analysis of the B7-H3 promoter regions using luciferase assay to accurately identify the promoter DNA sequences. This step was crucial for pinpointing the exact regulatory regions of interest. Secondly, we included 500 bp random DNA sequence as a negative control in our experiments. This control allowed us to differentiate between specific protein-DNA interactions and non-specific binding, thus enhancing the specificity of our findings. Additionally, we employed semi-quantitative proteomic data analysis to further refine our results. By comparing the proteins bound to the B7-H3 promoter region in CSCs with those bound to the negative control DNA and in non-CSC cell lines, we were able to filter out non-specific proteins and focus on the genuine regulatory factors. These methodological refinements enabled us to overcome the inherent limitations of the DAP-MS technique and confidently identify the transcription factors regulating B7-H3 expression.

Using these refined methodologies, we identified several key TFs (XRCC5, PARP1, DDB1, RPA1, and RPA3) regulating B7-H3 expression. The identification of these TFs is significant for several reasons. XRCC5, also known as Ku80, is primarily recognized for its role in DNA repair and maintaining genomic stability.^38^^,^^39^ Its involvement in B7-H3 regulation suggests a potential link between DNA repair mechanisms and immune evasion in cancer cells, implying that CSCs might leverage DNA repair pathways not only for survival but also to modulate immune checkpoints, thereby enhancing their ability to evade immune surveillance.

PARP1 is another DNA repair protein that has gained considerable attention in cancer research, particularly due to the development of PARP inhibitors as cancer therapeutics.^38^ The role of PARP1 in regulating B7-H3 expression adds a new dimension to its function, indicating that PARP1 might be involved in modulating immune checkpoint molecules in addition to its well-established roles in DNA repair. This dual functionality could provide a rationale for combining PARP inhibitors with immune checkpoint therapies to enhance anti-tumor efficacy.

DDB1 (damage-specific DNA binding protein 1) is a key component of the ubiquitin-proteasome system and plays a crucial role in numerous cellular processes, including DNA repair, cell-cycle regulation, and apoptosis. Our study is the first to identify DDB1 as a transcriptional regulator of B7-H3, revealing a novel function for this protein beyond its established roles in DNA repair. Inhibition of DDB1 with NTZ significantly reduced B7-H3 expression in CSCs, which corresponded with a decrease in tumor sphere formation and cell migration. This suggests that DDB1 plays a critical role in maintaining the stemness and tumorigenic potential of CSCs by regulating B7-H3 expression. The specific binding of DDB1 to the B7-H3 promoter region was confirmed through DNA pull-down assays, underscoring its direct regulatory influence.

RPA1 and RPA3, subunits of the replication protein A complex, are essential for DNA replication and repair.^40^^,^^41^ Their involvement in B7-H3 regulation further underscores the intricate link between DNA repair pathways and immune checkpoint regulation. The RPA complex is crucial for maintaining genomic integrity,^42^^,^^43^ and its role in B7-H3 expression suggests that disrupting RPA function could impact both DNA repair and immune evasion mechanisms in cancer cells.

The discovery of these TFs provides a deeper understanding of the regulatory networks governing B7-H3 expression in CSCs. It also opens up new therapeutic avenues. For instance, targeting these TFs could potentially downregulate B7-H3 expression, thereby reducing immune evasion and enhancing the effectiveness of existing cancer therapies. Moreover, the dual role of these TFs in both DNA repair and immune regulation presents an opportunity to develop combination therapies that target multiple cancer cell vulnerabilities simultaneously.

Among these TFs, our study is the first to identify DDB1 as a transcriptional regulator of B7-H3, revealing a novel function for this protein beyond its established roles in DNA repair. Inhibition of DDB1 significantly reduced B7-H3 expression, which in turn suppressed CSC properties such as tumor sphere formation and cell migration. This suggests that DDB1 plays a critical role in maintaining the stemness and tumorigenic potential of CSCs by regulating B7-H3 expression. The specific binding of DDB1 to the B7-H3 promoter region was confirmed through DNA pull-down assays, underscoring its direct regulatory influence.

In summary, our study explores the TFs involved in the regulation of B7-H3 expression and highlights the potential of targeting these newly identified TFs to disrupt CSC maintenance. Integrating these findings into therapeutic strategies could pave the way for more effective and targeted cancer therapies, particularly for cancers characterized by high B7-H3 expression and CSC prevalence. Although our findings provide valuable insights into the regulatory mechanisms of B7-H3 expression, further research is warranted to fully understand the functional roles of these TFs and their impact on B7-H3 regulation and CSC properties.

### Limitations of the study

Although this study provides key insights into the transcriptional regulation of B7-H3 in CSCs, several limitations should be acknowledged. First, although DAP-MS effectively identified TFs associated with B7-H3 expression, the approach may capture non-specific interactions or overlook transient binding events, necessitating further validation through complementary techniques such as ChIP-seq. Second, our findings are based on *in vitro* models, which, although useful for controlled analysis, may not fully represent the complexity of B7-H3 regulation in the tumor microenvironment. Future studies should investigate the dynamics of B7-H3 transcriptional regulation in physiological and disease-relevant contexts. Lastly, although our study identifies key TFs involved in B7-H3 expression, their broader role in CSC biology and interaction with other regulatory networks remains to be fully elucidated. Further research is needed to dissect how these TFs integrate with signaling pathways that influence CSC maintenance and tumor progression.

## Resource availability

### Lead contact

Further inquiries and resource requests should be directed to the lead contact, Dr. Kristine M. Kim (kmkim@kangwon.ac.kr).

### Materials availability

No materials such as reagents or other products were generated in this study.

### Data and code availability

• All data reported in this paper will be available upon request by contacting the lead contact.
• The mass spectrometry proteomics data have been deposited in the ProteomeXchange Consortium via the PRIDE^44^ partner repository under the dataset identifier PXD054584.
• This manuscript did not generate any original code.
• Any additional information required to reanalyze the data reported in this study is available upon request from the lead contact.

## Acknowledgments

This research was funded by the 10.13039/501100003725National Research Foundation of Korea (NRF) grant funded by the Korea government MIST (RS-2023-00245859 to E.C.Y., and NRF-2022M3E5F2028363, NRF-2022R1F1A1074094, and RS-2025-00553130 to K.M.K.). This research was supported by the BK21 FOUR funded by the Ministry of Education (MOE, Korea) and the research grant of 10.13039/501100002507Kangwon National University in 2022.

## Author contributions

E.C.Y. and K.M.K. conceptualized the project, designed experimental approaches, and supervised the project; Y.R.S., H.B.K., and H.R.J. performed and analyzed the LC-MS/MS data; Y.R.S. and H.B.K. generated plasmid DNA constructs; H.B.K. and S.M.H. performed DNA binding assay; Y.R.S. and E.H.K. performed cell proliferation assay; and E.C.Y. and K.M.K. interpreted the data and wrote the manuscript with input from all authors.

## Declaration of interests

The authors declare no conflict of interest.

## STAR★Methods

### Key resources table

**Table:** 

REAGENT or RESOURCE	SOURCE	IDENTIFIER
**Antibodies**		

Mouse monoclonal anti-Ku86	Santa Cruz Biotechnology	Cat#sc-5280; RRID:AB_672929
Mouse monoclonal anti-PARP1	Santa Cruz Biotechnology	Cat#sc-8007; RRID:AB_628105
Mouse monoclonal anti-DDB1	Santa Cruz Biotechnology	Cat# sc-376860; RRID:AB_2894825
Mouse monoclonal anti-RPA1	Santa Cruz Biotechnology	Cat# sc-48425; RRID:AB_628225
Mouse monoclonal anti-RPA3	Santa Cruz Biotechnology	Cat# sc-56770; RRID:AB_785534
Goat polyclonal anti-B7H3	R&D Systems	Cat# AF1027; RRID:AB_354546
Rabbit monoclonal anti-GAPDH	Cell Signaling technology	Cat# 2118S
B7-H3 Antibody (F-11) Alexa Fluor® 647	Santa Cruz Biotechnology	Cat# sc-376769; RRID:AB_2943305

**Chemicals, peptides, and recombinant proteins**		

Polyethylenimine	Sigma-Aldrich	408719-100ML; CAS: 25987-06-8
MDA-MB453 Cell Avalanche™ Transfection Reagent	EZ biosystems	EZT-MDAM-3
Lipofectamine™ RNAiMAX Transfection Reagent	Invitrogen™	13778075
Lipofectamine 3000 Transfection Reagent	Invitrogen™	L3000075
Nitazoxanide	Selleckchem	S1627; CAS: 55981-09-4

**Critical commercial assays**		

Dual-Luciferase® Reporter Assay System	Promega	E1910
PrimeScript™ 1st strand cDNA Synthesis Kit	Takara	6110A
TB Green® Premix Ex Taq™	Takara	RR420A

**Deposited data**		

Proteomics raw data	ProteomeXchange Consortium; PRIDE	PXD054584

**Experimental models: Cell lines**		

PANC-1	KCLB	KCLB# 21469
HCT116	KCLB	KCLB# 10247
Hela	KCLB	KCLB# 10002
HEK293	KCLB	KCLB# 21573
CSC-like MDA-MB453, non-CSC-like MDA-MB453	University of Pittsburgh Medical Center	

**Oligonucleotides**		

Primer: B7H3 Forward: AGCTGTGAGGAGGAGA ATGC; Reverse: TGCTGTCAGAGTGTTTCAGAGG	This paper	N/A
Primer: XRCC5 Forward: AAAGAGTTGGGTAGT TGTGGACGCA; Reverse: TCCATAGCGGAACC CTTGAATAG	This paper	N/A
Primer: PARP1 Forward: CCTGATCCCCCAC GACTTT; Reverse: GCAGGTTGTCAAGCATTTC	This paper	N/A
Primer: DDB1 Forward: AACAGAGTGGCGAGA GCATT; Reverse: TCAATGACATGCAGCTCCTC	This paper	N/A
Primer: DDB2 Forward: TCAAGGACAAACCC ACCTTC; Reverse: GTGACCACCATTCGGCTACT	This paper	N/A
Primer: RPA1 Forward: CGAGTCTCTGATTTCGGTG GAC; Reverse: GGCTTGTCCTTCTGCGTCAAAC	This paper	N/A
Primer: RPA3 Forward: AAGCCTGTCTGCTTCGTA GGGA; Reverse: CGGTTACTCTTCCAACCACTTCC	This paper	N/A

**Recombinant DNA**		

p3xFLAG-CMV™-7.1 expression vector	Sigma-Aldrich	Cat#E7533
pGL3 Basic Vector	Debrya Groskreutz	Addgene Plasmid #212936
p3xFlag-myc-CMVTM-26-XRCC5	Ewha Womans University	N/A
p3xFlag-CMV-PARP1	Sejong University	N/A

**Software and algorithms**		

Perseus 2.0.11.0 software	N/A	https://maxquant.net/perseus/
Gene Ontology (version 14)	PANTHER	https://geneontology.org/
DAVID (DAVID Bioinformatics Resources 6.8)	Xuezhi Zhou et al.	
Proteome Discoverer (version 2.4)	Thermo Scientific	
GraphPad Prism (9)	GraphPad Software, San Diego, CA, USA	www.graphpad.com
ImageJ		https://imagej.nih.gov/ij/

### Experimental model and study participant details

#### Cell lines

The human pancreatic cancer cell line (PANC-1; KCLB No. 21469), human colon cancer cell line (HCT116; KCLB No. 10247), human cervical cancer cell line (HeLa; KCLB No. 10002), and human embryonic kidney cell line (HEK293; KCLB No. 21573) were purchased from the Korean Cell Line Bank (KCLB). Cancer stem cell-like cells (CSCs) derived from MDA-MB-453 cells and non-cancer stem cell (NCSCs) were obtained from the University of Pittsburgh Medical Center.^45^ All cell lines were cultured in Dulbecco’s Modified Eagle’s Medium (DMEM) supplemented with 10% fetal bovine serum (FBS), penicillin (100 U/mL), and streptomycin (100 μg/mL), and maintained at 37°C in a 5% CO_2_ atmosphere. All cell lines used in this study were tested for mycoplasma contamination using PCR-based mycoplasma detection assays, and only mycoplasma-free cultures were used in experiments. The use of cell lines obtained from commercial sources and research institutions adhered to institutional guidelines and ethical standards.

### Method details

#### Plasmid construction and transfection

Human DDB1, RPA1 and RPA3 were cloned into the p3xFLAG-CMV-7.1 expression vector (Sigma-Aldrich, USA). The human B7-H3 promoter was cloned into pGL3 luciferase reporter vector. B7-H3 promoter DNA was generated by polymerase chain reaction (PCR). All construct sequences were confirmed by DNA sequence analysis. p3xFlag-myc-CMVTM-26-XRCC5, p3xFlag-CMV-PARP1 were a gift from Ewha Womans University and Sejong University.^46^^,^^47^ Cells were transfected with FLAG-tagged XRCC5, PARP1, DDB1, RPA1, RPA3 and truncated pGL3-B7-H3 promoter constructs using polyethylenimine (Sigma-Aldrich, St. Louis, MO) or Lipofectamine 3000 and MDA-MB453 Cell Avalanche Transfection Reagent (EZ biosystem, MD, USA) according to manufacturer instructions.

#### Luciferase reporter assay

CSCs were seeded (3 × 10^5^ cells) in 6-well plates and incubated for 24hrs before transient transfection with 5 μg of luciferase plasmid using MDA-MB453 Cell Avalanche Transfection Reagent (EZ biosystem, MD, USA) following the manufacturer’s protocol. After 48 h transfection, luciferase activities of the cell lysates were measured using a Dual-Luciferase Reporter Assay System (Promega, Madison, WI, USA) following the manufacturer’s instructions. The data were normalized for transfection efficiency by dividing firefly luciferase activity with that of Renilla luciferase.

#### DNA affinity purification assay

The sequences of −1000 to -1bp 5′-flanking region were amplified by PCR and tagged with biotin. The biotinylated promoter was incubated with 1mg ∼ protein extracted from CSCs and non-CSCs using Nucleus fractionation method and then gentle rotation for over-night at 4°C. The biotin-labeled promoter was bound with Pierce Streptavidin Agarose (Thermo Scientific) for 1 h at room temperature. The bound beads-promoter protein complexes were then washed three times with ice-cold PBS eluted with SDS-PAGE sample buffer.

#### Sample preparation, SDS-PAGE and in-gel digestion

Cells were lysed in NP-40-based lysis buffer (50mM Tris-HCl, 150mM NaCl, 1% NP-40, 1mM EDTA, pH 7.4) supplemented with broad-spectrum protease inhibitor cocktail and centrifuged at 14,000 rpm for 15 min. Proteins were denatured and reduced in SDS-PAGE sample buffer by boiling at 95°C for 10 min. The protein samples were than fractionated on 4–12% gradient Bolt Bis-Tris gel (Invitrogen, MA, USA). The gel was stained with Instant Blue (Sigma-Aldrich, MO, USA) and destained with water. In-gel tryptic digestion was conducted following the general protocol.^48^ Each gel lane was cut into 10 slices and diced into pieces on a glass plate. The excised gel pieces were washed and destained three times for 20 min with 50% (v/v) acetonitrile (ACN) (JT Baker, MA, USA) in 25 mM ammonium bicarbonate (NH_4_HCO_3_) (Sigma-Aldrich) and dried. Gels were reduced by 25 mM DTT in 100 mM NH_4_HCO_3_ at 60°C for 1 h and alkylated with freshly prepared 55 mM iodoacetamide (IAA) (Sigma-Aldrich) in NH_4_HCO_3_ in the dark for 45 min. The gel particles were shrinked by 100% ACN, completely dried using the speed-vac concentrator, saturated with 12.5 ng/μL trypsin (Promega, Madison, WI, USA) in 50 mM of NH_4_HCO_3_ on ice for 45 min, and digested at 37°C for over-night. Peptides were extracted after incubation with 10% formic acid (FA) for 15 min on ice, and another extra extraction step was performed after incubation with 50% (v/v) ACN/0.1% (v/v) FA and 80% (v/v) ACN/0.1% (v/v) FA serially. The extracted peptides were dried under a concentrator and stored at −20°C until used.

#### Mass spectrometry analysis and database search

The extracted peptides from gel fractions were suspended in 40 μL of Solvent A (0.1% FA in water) and 10 μL of suspended peptides were injected from an autosampler into a trap column (uPAC RP C18 column 1 cm, PharmaFluidics, Ghent, Belgium) with isocratic gradient of 2% Solvent B (0.1% FA in ACN) for 8 min at a flow rate of 10 μL/min. After that, sample was loaded onto an analytical column (uPAC RP C18 column 50 cm, PharmaFluidics, Ghent, Belgium) through valve change and separated with a multi-step gradient of 5%–35% B for 60 min at a flow rate of 300 nL/min. MS spectra were recorded on a Q-Exactive hybrid quadrupole-Orbitrap mass spectrometry (Thermo Fisher Scientific, Bremen, Germany) coupled with Dionex Ultimate 3000-RSLCnano-system (Dionex, Sunnyvale, CA, USA). Standard mass spectrometric condition of the spray voltage was set to 1.9kV and the temperature of the heated capillary was set to 250°C. The full scans were acquired in the mass analyzer at 400–1400 m/z with a resolution of 70,000 and the MS/MS scans were obtained with a resolution of 17,500 by normalized collision energy of 27 eV for high-energy collisional dissociation (HCD) fragmentation. The advanced gain control (AGC) target was 3 × 10^6^, maximum injection time (IT) was 100 ms, and the isolation window was set to 2 m/z. the Q-Exactive was operated in data-dependent mode with one survey MS scan followed by ten MS/MS scans, and the duration time of dynamic exclusion was 30 s. Collected MS/MS raw data were searched against the decoy UniProt human database for the estimation of the false discovery rate (FDR) with the SEQUEST data analysis program in the Proteome Discoverer (Thermo, version 2.4) search platform. Precursor and fragment ion tolerance were set to 10 ppm and 0.02 Da, respectively. Trypsin was selected as enzyme with a maximum allowance of up to two missed cleavages. Fixed modifications for carbamidomethyl-cysteine (+57.0215Da) and variable modification for methionine oxidation (+15.9949 Da) were used. Label-free quantification was conducted using the Minora feature detector in Proteome Discoverer. The relative quantification was determined based on the area under the curve (AUC) of the precursor ions. All proteins with a ProteinProphet probability up to 99% with minimum two peptides and a PeptideProphet probability up to 95% were identified using Scaffold (version 4.8.9; Proteome Software, Portland, OR, USA).^49^^,^^50^

#### Quantitative analysis of identified proteins

The protein tables generated in Proteome Discoverer were exported and then imported into Perseus 2.0.11.0 software (https://maxquant.net/perseus/) for normalization and statistical analysis. Protein abundances were normalized by first dividing each row by its median value and then dividing each column by the median value of proteins in that column. Following normalization, the data were log2-transformed. Log2 fold changes were calculated by subtracting the average log2-transformed values between two groups. A Student’s two-sample t-test was used to determine *p*-values. Significance Analysis of Microarrays (SAM) and permutation-based FDR estimation were performed to obtain q-values. Differentially expressed proteins identified in each comparative analysis were analyzed by Gene Ontology (GO) analysis, utilizing the Database for Annotation, Visualization, and Integrated Discovery (DAVID) online database (DAVID Bioinformatics Resources 6.8).

#### siRNA-mediated silencing

siRNA oligonucleotides for XRCC5, PARP1, DDB1, DDB2, RPA1, RPA3, and B7-H3 were purchased from Bioneer (Daejeon, Korea). Cells (0.3 × 10^5^cells) were seeded in 6-well plates for each well and allowed to grow for 24 h. Cells were transfected with Lipofectamine RNAiMAX (Invitrogen) and siRNA (XRCC5, PARP1, DDB1, DDB2, RPA1, RPA3, and B7-H3) or scrambled siRNA (siRNA-negative control, NC), Opti-MEMTM media: 300 μL, Lipofectamine RNAiMAX: 9 μL, 30pmol siRNA (for each well) for 24 or 48 h.

#### Immunoblot analysis

For immunoblotting, 20–40 μg of protein extracts were boiled and separated by 4–12% SDS-polyacrylamide gel electrophoresis (SDS-PAGE). Proteins were transferred to polyvinylidene difluoride membranes which was activated by methanol, blocked for 1h in Tris-buffered saline (TBS) containing 0.1% Tween 20 and 5% (w/v) dry skim milk powder, and incubated overnight with the indicated primary antibodies. The membranes were then incubated for 1h with an HRP-conjugated secondary antibody (rabbit-HRP, mouse-HRP (Afrontier, Seoul, Korea), goat-HRP (R&D Systems)). Proteins were visualized with a chemiluminescence detection system (cytiva). Band intensities were quantified using Amersham Imager 680 Analysis Software and results are expressed relative to the control condition.

#### RNA isolation and quantitative real-time PCR

Total RNA from cancer cells was isolated using TRIzol (Favorgen Biotech, Taiwan). The complementary DNA (cDNA) preparation was preformed using cDNA Reverse Transcription Kit (Takara, Japan). Quantitative real-time PCR (qPCR) was performed with the SYBR Green quantitative PCR kit (Takara, Japan) using the 7500 Real-Time PCR System. The primers used in experiment were as follows.

18s rRNA
  forward primer: 5′- GCTTAATTTGACTCAACACGGGA – 3’
  reverse primer: 5’ – AGCTATCAATCTGTCAATCCTGTC – 3’
GAPDH
  forward primer: 5’ – TGTAGACCATGTAGTTGAGGTCA – 3’
  reverse primer: 5’ – AGGTCGGTGTGAACGGATTTG – 3’
B7-H3
  forward primer: 5’ – AGCTGTGAGGAGGAGAATGC – 3’
  reverse primer: 5’ – TGCTGTCAGAGTGTTTCAGAGG – 3’
XRCC5
  forward primer: 5’ – AAAGAGTTGGGTAGTTGTGGACGCA – 3’
  reverse primer: 5’ – TCCATAGCGGAACCCTTGAATAG – 3’
PARP1
  forward primer: 5’ – CCTGATCCCCCACGACTTT – 3’
  reverse primer: 5’ – GCAGGTTGTCAAGCATTTC – 3’
DDB1
  forward primer: 5’ – AACAGAGTGGCGAGAGCATT – 3’
  reverse primer: 5’ – TCAATGACATGCAGCTCCTC – 3’
DDB2
  forward primer: 5’ – TCAAGGACAAACCCACCTTC – 3’
  reverse primer: 5’ – GTGACCACCATTCGGCTACT – 3’
RPA1
  forward primer: 5’ – CGAGTCTCTGATTTCGGTGGAC – 3’
  reverse primer: 5’ – GGCTTGTCCTTCTGCGTCAAAC – 3’
RPA3
  forward primer: 5’ – AAGCCTGTCTGCTTCGTAGGGA – 3’
  reverse primer: 5’ – CGGTTACTCTTCCAACCACTTCC – 3′

#### Flow cytometry

Cells were harvested, washed twice with FACS buffer (0.5% BSA and NaN_3_), and blocked with 3% BSA at 4°C for 30 min. Cells were stained with Alexa Fluor 647-conjugated B7-H3 at 4°C for 1 h. The instrument threshold was kept constant throughout the detection by BD LSRII (SORP) (BD Bioscience).

#### MTT assay

Cells were plated in cell culture plates and incubated in CO_2_ incubator. Treatment was given according to the experimental requirement. Further, at respective time points, MTT solutions from the Stock (final con. 5 mg/mL) was added and cells were incubated in CO_2_ incubator in the dark for 1 hr. The medium was removed, and Formazan crystals formed by the cells were dissolved using DMSO followed by transfer in 96 well plate. The absorbance was read at 570 nm using 630 nm as reference wavelength on a Multi-well plate reader (Biotech Instruments, USA). Reduced formazan quantification was done with Formazan standard.

#### Tumor sphere formation assay

Cells were seeded in adherent culture conditions. To perform sphere formation assays, cells were plated in Ultra-Low attachment multi-well plate (Corning Life Sciences, Tewksbury, MA, USA) at a concentration of about 1 × 10^4^ cells/mL of sphere-forming medium. After 4–7 days of growth, spheres were counted.

#### Wound healing assay

Cells were plated in 24-well plates. After cells were achieved confluence, wounds were scratched by dragging a sterile pipette tip across the monolayer. Photographs of the movement of cells into the scratch area were taken every 12 h until the scratch area had closed using microscope. Wound healing was then analyzed using ImgaeJ (Java) software (Rasband, NIH, Bethesda, MD, USA).

#### Transwell migration assay

Cell migration was measured using Transwell inserts (Corning Life Sciences, Tewksbury, MA, USA) according to the manufacturer’s instructions. Cells were seeded in transwell inserts at a concentration of 2 × 10^4^ cells/well. After 4 days of growth, migrated cells were measured using MTT assay.

### Quantification and statistical analysis

Statistical significance was determined by two-tailed Student’s t test (∗, *p* < 0.05; ∗∗, *p* < 0.01; ∗∗∗, *p* < 0.005; ∗∗∗∗, *p* < 0.001). All results were presented as the means ± SEM from at least three independent experiments.
