# Supplementary material for: Unveiling transcriptional mechanisms of B7-H3 in breast cancer stem cells through proteomic approaches
Source: iScience. 2025 Mar 14;28(4):112218. doi: 10.1016/j.isci.2025.112218 (PMC11995042; doi:10.1016/j.isci.2025.112218)
Supplement: Document S1. Figures S1–S5 [file mmc1.pdf]

iScience, Volume *28*

Supplemental information

Unveiling transcriptional mechanisms of B7-H3 in breast cancer stem cells through proteomic approaches

Yu Ri Seo, Han Byeol Kim, Hyeryeon Jung, Eunhee G. Kim, Sumin Huh, Eugene C. Yi, and Kristine M. Kim

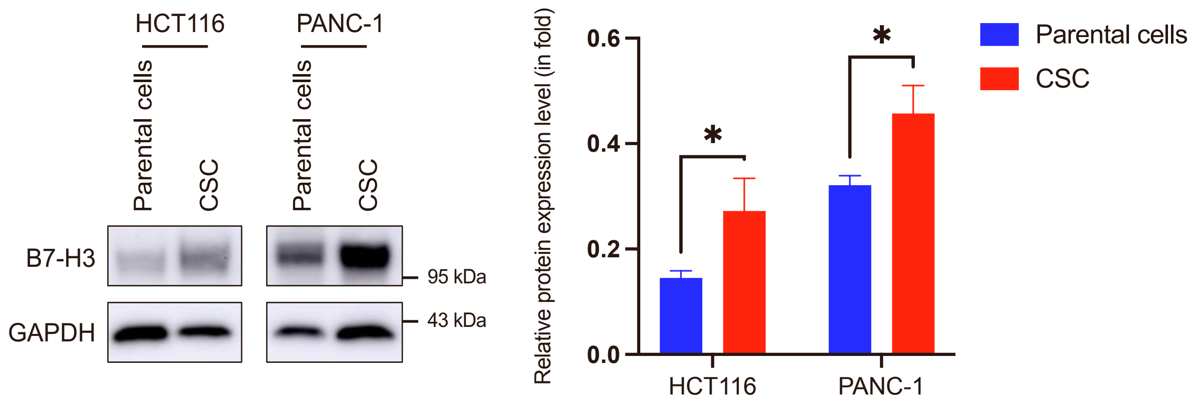

Figure S1. B7-H3 Expression in CSC Populations Across Various Cancer Cell Lines, related to Figure 1.

Western blot analysis showing B7-H3 expression in cancer stem cell (CSC) populations derived from HCT116 and PANC-1 cells, compared to their respective parental cell lines. GAPDH was used as a loading control. Data are representative of three independent experiments, with values expressed as mean ± SD. Statistical significance is indicated as follows: *P<0.05, **P<0.01, ***P<0.001.

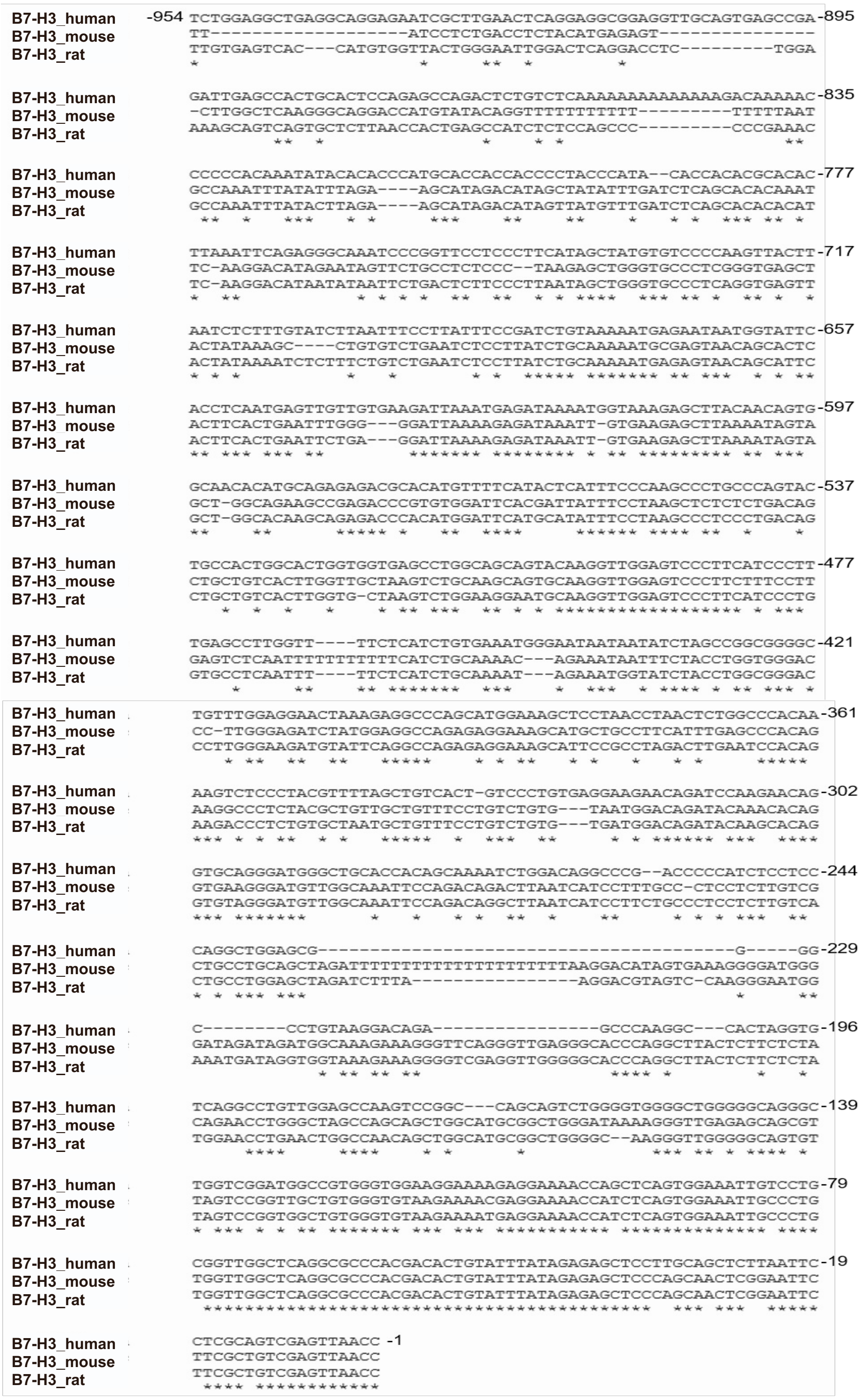

Figure S2. Homology comparison of minimal promoter region of B7-H3 across species, related to Figure 2.

Homology comparison of minimal promoter region of human B7-H3 gene with mouse and rat using Clustal Omega of EMBL-EBI. The gene names and species are provided. Conserved sequences are marked with an asterisk (*). The 1000bp upstream TSS sequence of the human B7-H3 gene shows significant conservation across species, indicating evolutionary conservation of regulatory elements.

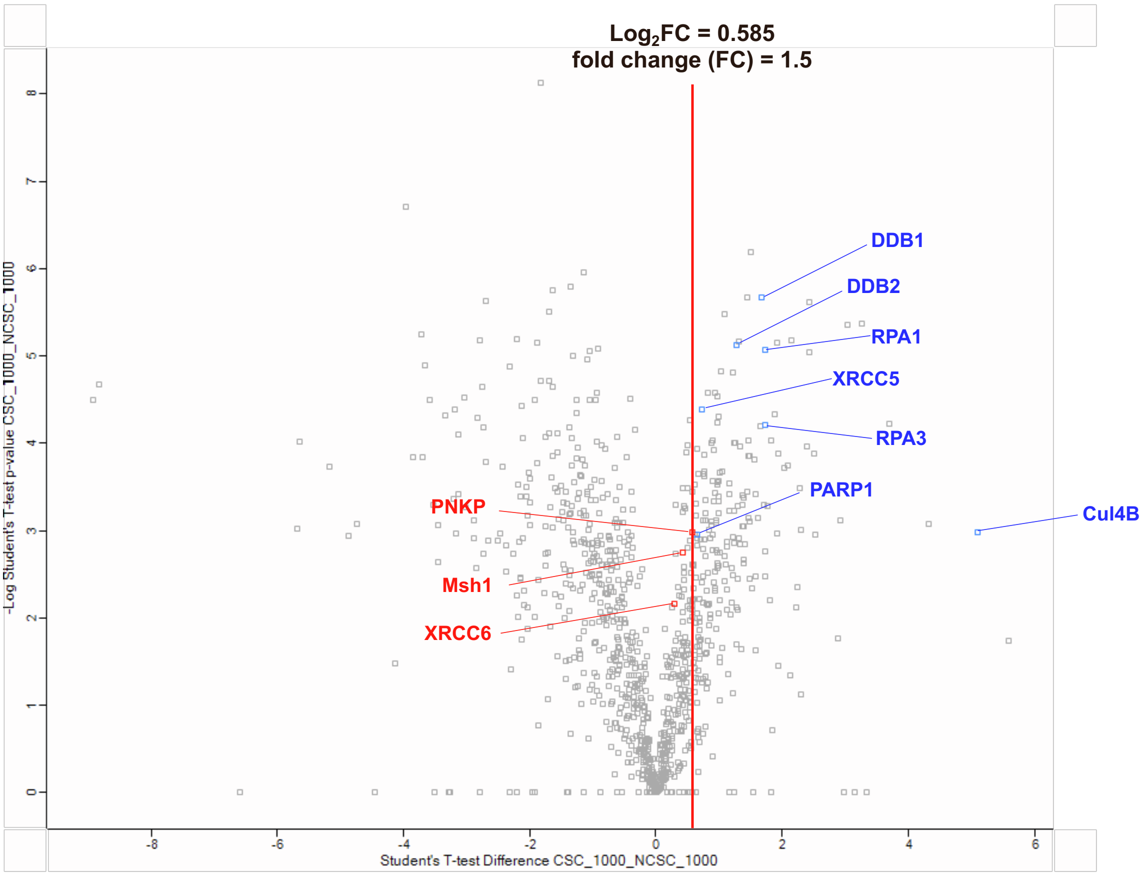

Figure S3. Volcano plot of differentially expressed proteins (DEPs), related to Figure 2.

Volcano plots showing the student's T-test difference and the adjusted Log2FC (fold change > 1.5). Blue dots represent significantly upregulated protein, while red dot indicated the downregulated proteins. The symbol-marked dots highlight the 10 proteins identified based on the Gene Ontology (GO) analysis. From the 10 proteins, seven were selected that bind to the 1000 bp B7-H3 promoter DNA in CSCs compared to NCSCs, with a fold change > 1.5.

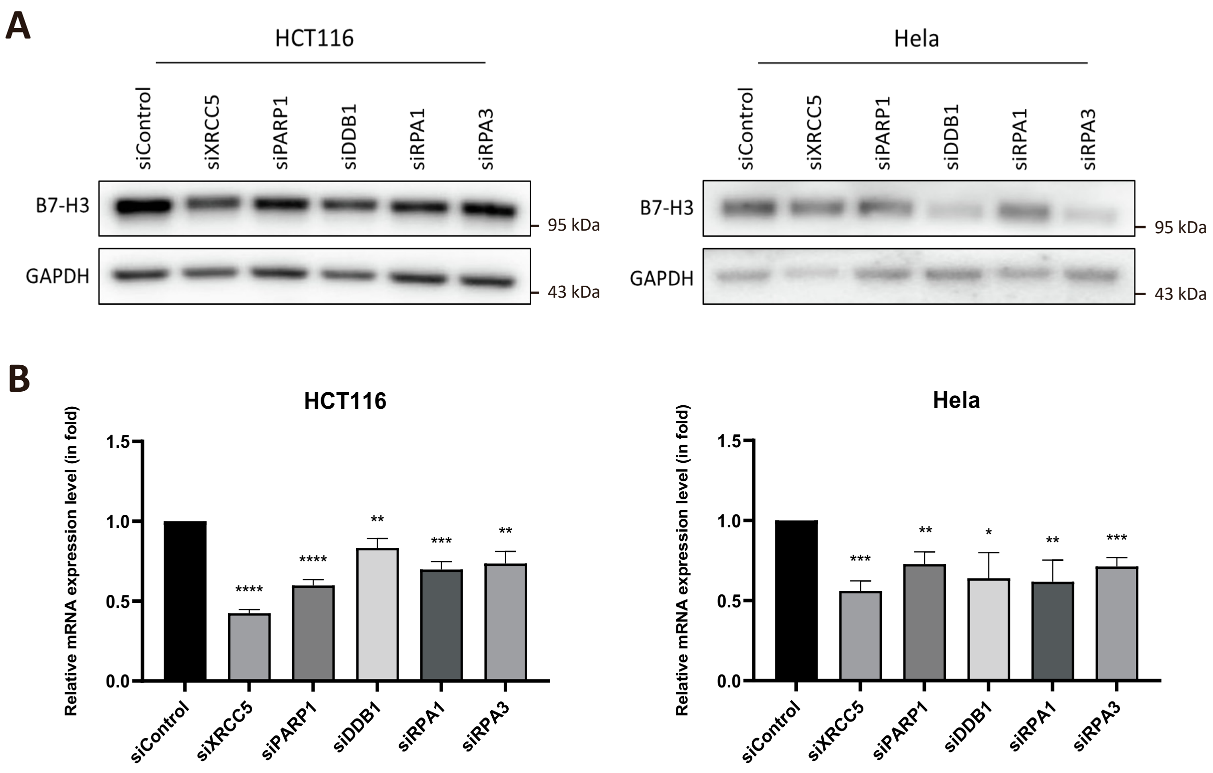

Figure S4. Regulatory functions of transcription factor (TF) candidates for B7-H3 expression in various cancer cell lines, related to Figure 3.

(A) Protein expression level of B7-H3 in HCT116 and Hela cell lines after transfection with active siRNA directed against the candidate proteins (siXRCC5, siPARP1, siDDB1, siDDB2, siRPA1, and siRPA3), siControl as negative control. (B) B7-H3 Protein expression level following siRNA knockdown of candidate TFs were measured by Western blot. GAPDH was used as a sample loading control. Representative results from three independent experiments are shown, and all the values were expressed as the means ± SD, *P<0.05, **P<0.01 and ***P<0.001. Knockdown of these TFs in both HCT116 and HeLa cell lines also led to reduced levels of B7-H3 protein and mRNA, reinforcing their regulatory roles in the expression of B7-H3.

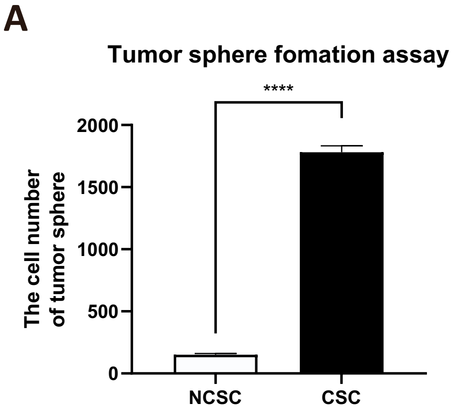

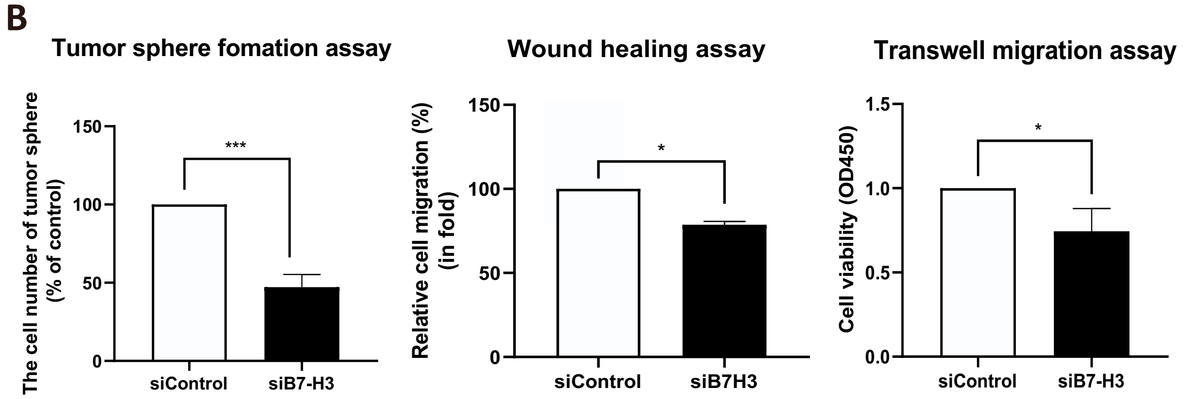

Figure S5. Effect of B7-H3 on stemness traits in CSCs, related to Figure 5.

(A) Enhanced tumor sphere formation capabilities of CSCs compared to non-cancer stem cells (NCSCs). (B) Effect of B7-H3 knockdown on stemness traits in CSCs, assessed through tumor sphere formation assay, wound healing assay, and transwell migration assay. Representative results from three independent experiments are shown. Values are expressed as means ± SD, with significance levels indicated as *P<0.05, **P<0.01, ***P<0.001, and ****P<0.0001. Knockdown of B7-H3 in CSCs led to a significant reduction in stemness traits, confirming B7-H3's role as a regulator of CSC properties.
